# Phage against the Machine: The SIE-ence of Superinfection Exclusion

**DOI:** 10.3390/v16091348

**Published:** 2024-08-23

**Authors:** Michael J. Bucher, Daniel M. Czyż

**Affiliations:** Department of Microbiology and Cell Science, University of Florida, Gainesville, FL 32611, USA

**Keywords:** superinfection exclusion, bacteriophage, fitness, antimicrobial resistance, *Pseudomonas aeruginosa*

## Abstract

Prophages can alter their bacterial hosts to prevent other phages from infecting the same cell, a mechanism known as superinfection exclusion (SIE). Such alterations are facilitated by phage interactions with critical bacterial components involved in motility, adhesion, biofilm production, conjugation, antimicrobial resistance, and immune evasion. Therefore, the impact of SIE extends beyond the immediate defense against superinfection, influencing the overall fitness and virulence of the bacteria. Evaluating the interactions between phages and their bacterial targets is critical for leading phage therapy candidates like *Pseudomonas aeruginosa*, a Gram-negative bacterium responsible for persistent and antibiotic-resistant opportunistic infections. However, comprehensive literature on the mechanisms underlying SIE remains scarce. Here, we provide a compilation of well-characterized and potential mechanisms employed by *Pseudomonas* phages to establish SIE. We hypothesize that the fitness costs imposed by SIE affect bacterial virulence, highlighting the potential role of this mechanism in the management of bacterial infections.

## 1. Introduction

Bacteriophages (phages) are obligate intracellular parasites, or viruses, of bacteria [1,2]. They undergo two primary lifestyles, lytic and lysogenic [3,4], and a less studied chronic cycle [5]. In the lytic lifecycle, phages bind to receptors on their host, inject their genetic material, hijack host replication machinery, and replicate themselves. At the final stage of this cycle, the lytic phage lyses the bacterial cell, killing it and releasing numerous phages that infect new hosts. Conversely, the lysogenic lifecycle begins in a similar fashion; however, after infection, lysogenic phages do not lyse their host. Instead, they integrate their genetic material into the host genome or remain in the cell as plasmids. This allows them to remain dormant and only replicate their DNA as the bacterial DNA replicates. Then, under specific conditions, these integrated phages, referred to as prophages, can become activated to begin the lytic lifecycle [6]. In the chronic cycle, virions, primarily filamentous phages, are produced and continuously released from the host cell without cell lysis or death [5,7].

Superinfection exclusion (SIE) describes the process by which viruses, particularly their prophage states, can inhibit the coinfection of their host cell by other similar phages. Typically, integrated prophages are maintained in a lysogenic state, where their lytic development is blocked by a repressor protein. However, under certain conditions, such as superinfection by another phage, specific prophage genes can become active even in the presence of a repressor [8,9]. Under superinfection conditions, activated prophage genes encode proteins that prevent the successful infection and replication of other phages through a variety of different mechanisms described in this review.

In nature, competition between organisms is one of the most important drivers of behavior affecting evolution and ecological dynamics [10]. Phages are not exceptions to this rule and, similarly to bacteria, they have developed mechanisms to outcompete other phages [11]. SIE represents one of the tactics employed by viruses to enhance their survival and replication efficiency. Once a phage finds its host, it can leverage the SIE mechanism to prevent coinfection of its host by other similar phages. Many of the mechanisms facilitating SIE involve direct changes at the surface of the bacterial host [12,13,14,15,16,17,18]. Consequently, even small modifications in surface components can have a profound impact on bacterial fitness, survival, and virulence [19].

*Pseudomonas aeruginosa* is an opportunistic pathogen that is ubiquitously present in the environment [20]. It is a motile, Gram-negative species that is known for its resistance to many antibiotics [21], including many clinically important classes such as β-lactams, quinolones, aminoglycosides, and polymyxins [22]. *P. aeruginosa* is a member of the ESKAPEE pathogens, a group of highly antibiotic-resistant bacteria that are the leading cause of hospital-acquired infections, which places them on the World Health Organization (WHO) global priority pathogen list [23,24]. Due to the high levels of antibiotic resistance and the severity of *P. aeruginosa* infections, phage therapy has emerged as a promising alternative to traditional antibiotics in the treatment of these infections [25,26,27,28]. There have been numerous cases of successful phage therapy against *P. aeruginosa*; as such, it is being more widely considered as a viable antimicrobial strategy [29,30,31]. However, phage therapy has several potential caveats that must be evaluated prior to implementation, including the relative ease by which bacteria develop resistance to phages, neutralizing antibody response, the difficulties associated with large-scale production of phages, and the selection of phages with genetic backgrounds that lack undesirable genes [32,33]. While the above-mentioned constraints that limit the broad use of phage therapy have been extensively studied and can be successfully addressed, the influence of SIE on *P. aeruginosa*, or other bacterial pathogens, has not been evaluated as an additional consideration when choosing suitable phages for therapeutic use. The following review focuses on the SIE mechanisms employed by *P. aeruginosa* phages and their implications in bacterial fitness and virulence.

## 2. Prophage Proteins Are Important for Superinfection Exclusion across Many Species

Most well-described SIE mechanisms rely on phage proteins. The primary mechanisms by which phages initiate SIE are the inactivation of phage receptors or the prevention of phage DNA injection and translocation [34]. Phage receptor inactivation typically involves the modification or complete assembly obstruction of important cell surface components essential for phage attachment and entry [12,35]. The prevention of phage DNA injection and translocation occurs through the sequestration of DNA at the surface of the bacterium or in a location where DNA transcription is impossible, such as within the periplasm of Gram-negative bacteria [36,37]. The inactivation of receptors is the most described anti-phage mechanism in the literature. However, it remains unclear whether the frequency of this mechanism is attributed to the relative ease of detecting physical changes in bacterial structures compared to the sequestration or inactivation of viral DNA, or if it is indeed the most frequently employed tactic in phage competition.

SIE is primarily mediated by phages, which could reasonably be explained when considering the significant competitive advantage these mechanisms confer to the phage. Due to the essential involvement of phage proteins in these processes, bacteria likely have a limited role, if any, in the initiation of SIE. Furthermore, the role of phage proteins might limit the ability of bacteria to evolve and suppress this phenomenon. Consequently, it is conceivable that SIE mechanisms impose host fitness impairment that resists elimination due to these evolutionary constraints.

## 3. *Pseudomonas* Filamentous Phage Pf Proteins Lead to Deactivated Type IV Pilus to Achieve Superinfection Exclusion

Specific *P. aeruginosa* phages have evolved distinct strategies for SIE, one of which involves the inactivation of the Type IV Pilus (T4P) by the *Pseudomonas* phage Pf4. Pf phages are filamentous and commonly found in *P. aeruginosa* isolates [38,39,40]. Among them, the Pf4 phage is a specific variant found in *P. aeruginosa* PAO1 [40]. Pf4 commonly undergoes a lysogenic lifecycle, integrating its genetic material into the bacterial genome upon infection [40]. Similarly to other filamentous phages, Pf4 can be excised from *P. aeruginosa* without disruption of the host [41,42]. This unique mechanism allows the Pf4 phage to accumulate and reinfect additional hosts without killing any cells [42]. Unlike many other filamentous phages, Pf4 does not contain any membrane channel-forming proteins, and it has been hypothesized that Pf4 may utilize host secretion systems for this extrusion pathway [43]. Pf4, however, can kill *P. aeruginosa* via superinfection, where multiple virions enter the same host, inducing cell lysis from within [41,44]. As a result, Pf4 has developed a method to prevent superinfection in *P. aeruginosa* to gain a competitive advantage over other virions and to maintain its cooperative exchange with the bacterial host [45].

SIE in *P. aeruginosa* primarily occurs through the inactivation of the T4P structure by Pf4, representing the most well-described SIE mechanism in this species [45]. The T4P serves as the receptor for Pf4, making it the target for SIE [42,46]. In *P. aeruginosa*, the T4P is essential for twitching motility [47,48,49] and is an important contributor to bacterial virulence across species [50]. Additionally, the T4P facilitates adherence and DNA uptake [51], while serving as a common receptor for various phages, not limited to the Pf family alone [52,53,54]. The T4P structure consists of thousands of copies of a single major subunit, making up the major pilus structure [55], and several minor subunits that have supportive functions in assembly and retraction [56]. The diversity of structures and the complexity of their interactions offer numerous potential targets for disruption by phages.

**Figure 1:**
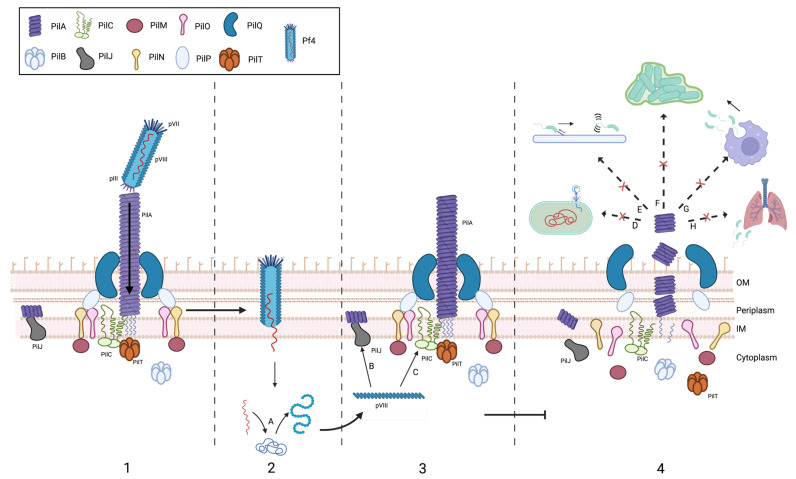
Simplified mechanism for *Pseudomonas* phage Pf4 superinfection exclusion and the proposed associated fitness effects. Superinfection exclusion begins with previous Pf4 infection and integration to the host genome. (**1**) Host receptor binding: the *P. aeruginosa* host receptor utilized by Pf4 is the T4P major pilin PilA (purple). After binding, the filamentous phage is pulled through the outer membrane via T4P retraction, mediated by the PilT (orange) ATPase. Next, the filamentous phage is brought through the periplasm and into the inner membrane (IM) via several other proteins not included in the figure. (**2**) DNA injection and integration: the phage then injects its linear ssDNA into the cytoplasm where it can integrate into the bacterial genome (A) and has its components synthesized utilizing host transcriptional and translational machinery. (**3**) Protein interaction during superinfection: under conditions of superinfection, the Pf4 phage protein pVIII interacts with (B) PilJ and (C) PilC, components of the T4P. (**4**) Impact on T4P assembly and virulence: these interactions prevent the complete biosynthesis and assembly of the T4P, likely affecting diverse virulence mechanisms, including (D) superinfection exclusion, (E) loss of twitching motility, (F) reduction in biofilm production, (G) reduced capacity for immune evasion, and (H) tissue invasion.

Several Pf4 phage proteins are involved in mediating the SIE process, including pVII, pVIII, and pIII [45]. Among these, the pVII minor capsid structural protein is essential for T4P inactivation (Figure 1) [45]. It has been shown that the pVII protein interacts with the cytoplasmic domain of the PilC accessory protein and PilJ chemotaxis protein of the T4P [45]. PilC, an inner membrane protein, is responsible for T4P assembly in *P. aeruginosa* [57], while PilJ is necessary for the complete assembly of the T4P [58]. Although the specific interaction between the pVII protein and PilC and PilJ is not well understood, it has been established that high levels of pVII lead to a significant reduction in Pf4 phage adsorption and the elimination of twitching motility in *P. aeruginosa* [45].

## 4. Additional *Pseudomonas* Phage Proteins May Lead to Superinfection Exclusion through the Inhibition of the Type IV Pilus and Other Essential Phage Receptors

Further studies have uncovered several additional phage proteins involved in disrupting phage receptors in *P. aeruginosa*. These additional studies underscore that phage-mediated receptor modifications as a mechanism for SIE are not limited to a single phage–host interaction. The wide range of mechanisms targeting the same surface structural elements emphasizes the importance of these receptors as targets for many phages.

Examples of such additional mechanisms that mediate SIE include the *Pseudomonas* phage D3112 phage-encoded accessory protein Gp05 and the *Pseudomonas* phage DMS3-encoded quorum sensing anti-activator protein Aqs-1 [13,14], which both inactivate PilB, an ATPase that provides the energy necessary for the complete assembly of the T4P [57,59]; therefore, inactivation of the PilB ATPase prevents assembly of the T4P [13,14]. Additionally, the *Pseudomonas* phage vB_Pae_QDWS phage-encoded Gp21 protein reduces the expression of PilA, the major pilin subunit, and the PilB ATPase [15]. Collectively, phage proteins facilitate SIE by modulating bacterial receptors, affecting their concentration, assembly, and functional levels [45].

*Pseudomonas* phage D3 has also been shown to alter its bacterial receptor to prevent superinfection by similar phages. The cellular receptor for D3 is the lipopolysaccharide (LPS). Upon infection of its host, *Pseudomonas* phage D3 O-acetylase (Oac) can acetylate a fucosamine residue on *P. aeruginosa* LPS, leading to a conversion event and ultimately changing the structure of the O-antigen region in a way that makes phage binding impossible [60,61]. Similar variations in this LPS serotype conversion mechanism have been identified in a number of other *Pseudomonas* phages [62,63,64,65,66]. Furthermore, some lytic *Pseudomonas* phages, such as C11 and K5, utilize the O-antigen of LPS as an essential receptor [67,68], suggesting the potential that more phages may target this specific receptor to facilitate SIE.

## 5. Other Pathogens of Importance Carry Mechanisms of Superinfection Exclusion with Close Functional Homologs in *P. aeruginosa*

**Figure 2:**
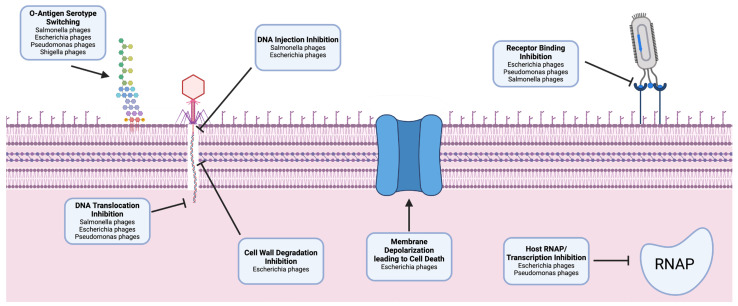
Overview of phage-induced superinfection exclusion (SIE) mechanisms across different bacterial species. Key mechanisms include the following: (1) O-Antigen serotype switching, where *Salmonella*, *Shigella*, *Escherichia*, and *Pseudomonas* phages alter the surface antigens of the host bacteria to evade phage recognition. (2) DNA injection inhibition by *Salmonella* and *Escherichia* phages prevents the introduction of competing phage DNA into the bacterial cytoplasm. (3) Receptor binding inhibition, a tactic used by *Escherichia*, *Pseudomonas*, and *Salmonella* phages to block or alter the structure of receptor sites, thus preventing attachment of subsequent phages. (4) DNA translocation inhibition, a process where *Salmonella*, *Escherichia*, and *Pseudomonas* phages stop the transport of foreign DNA into the host cell. (5) Cell wall degradation inhibition, employed by *Escherichia* phages to maintain cell wall integrity against enzymatic degradation by other phages. (6) Membrane depolarization leading to cell death as a defensive measure by *Escherichia* phages, causing host cell death upon superinfection. (7) Host RNA polymerase (RNAP)/transcription inhibition, a technique used by *Escherichia* and *Pseudomonas* phages to interfere with the host’s RNA polymerase, halting host transcription, and propagation of competing phages. The phage components and bacterial targets are represented schematically to depict the interactions and effects of SIE mechanisms.

Inactivation of phage receptors as a SIE strategy is not unique to *P. aeruginosa*. Similar tactics have been observed in other bacterial species, including *Salmonella enterica*, *Shigella flexneri*, and *Escherichia coli* (Figure 2, Table 1). The common use of similar receptors by diverse phages across multiple species underscores the importance of the exclusion mechanism. Such cross-species similarities in SIE strategies suggest that by studying the mechanisms in one phage–host system, we might gain insights into the tactics potentially employed by phages in other bacterial hosts.

A specific example of these cross-species similarities is demonstrated by the ability of *Salmonella* phage P22 to acetylate the O-antigen region of the LPS [17,69,70]. The P22 genome encodes a specific glycosyltransferase (gtr) operon, which contains genes for three unique enzymes, GtrA, GtrB, and GtrC. GtrA and GtrB are conserved membrane proteins, the flippase and bactoprenol glucosyl transferase, respectively. GtrC, however, is more variable and encodes a glycosyltransferase that serves as a mediator for the attachment of the glucose segment to the O-antigen [70]. The importance of this enzyme is emphasized by the specific modification of the *S. enterica* O-antigen, which serves as an essential receptor during P22 phage infection [71]. As a result of the aforementioned LPS modification, phages that utilize O-antigen as receptors fail to infect their hosts. Several other phages, including *Shigella* phages SF6 and SF101, encode proteins that alter the O-antigen portion of LPS in other bacterial species, most notably *Shigella flexneri* [72,73,74].

Additional examples of SIE mechanisms that block the adsorption of phages by receptor modification are observed across diverse species. Some of these mechanisms are likely conserved across genera due to the existence of functionally similar proteins. An example of such conservation is outlined by the inactivation of the FhuA receptor by the Cor protein found in *Escherichia* phages φ80 and N15 [75]. The FhuA receptor, a TonB-dependent ferric siderophore transporter, is a common phage receptor known for transporting phage DNA across membranes in multiple species [16,76]. Several functionally similar TonB-dependent siderophore transporters exist in *P. aeruginosa* [77].

Another example of a possible SIE mechanism used by *P. aeruginosa* phages comes from *Escherichia* phage T4, which utilizes the products of the *imm* and *sp* genes that prevent DNA injection by changing the stability of the translocation site and by preventing degradation of the peptidoglycan layer through lysozyme modulation, respectively [78].

Some other notable mechanisms that *Pseudomonas* phages might mimic include the ability of *Salmonella* phage P22 to inhibit viral DNA injection. It accomplishes this through an encoded inner-membrane protein, SieA, which blocks the passage of DNA through the periplasm, a crucial step that most *Pseudomonas* phages must successfully perform [36]. Moreover, the Lambda phage of *E. coli* is capable of excluding multiple phages from infection both by manipulating the metabolism of its host and by preventing transcription of superinfecting lambda phage genes [79,80,81,82,83,84]. Lambda phage-dependent metabolic alterations are facilitated by two unique proteins, RexA and RexB. These two phage-encoded proteins lead to the formation and activation of ion channels, resulting in membrane depolarization and the cessation of macromolecular synthesis, loss of several essential biological functions, and even cell death. Consequently, compromised cellular energetics make it impossible for infecting lytic phages to replicate, as they lack the necessary materials and assembly apparatus [85,86,87]. Superinfecting lambda phage gene transcription is inhibited by the phage CI repressor, which attenuates the expression of lytic phage genes, resulting in SIE and, ultimately, maintaining lambda phage lysogeny [84,88,89]. Furthermore, the T7 phage in *E. coli* utilizes phage-encoded proteins, Gp2 and Gp0.7, to inactivate the host RNA polymerase [90]. The phage encodes its own RNA polymerase that is not inactivated by Gp2 and Gp0.7 [91]. This mechanism prevents the successful replication of any superinfecting phages, as the T7 RNA polymerase exhibits exceptionally high specificity to the T7 promoter, thereby outcompeting any phage genomes awaiting transcription [92,93,94,95]. As a result, this SIE mechanism almost entirely prevents the replication of competing phages. Several *Pseudomonas* phages are known to carry T7-like RNA polymerases [96,97], and it has been shown that some *Pseudomonas* phages, such as φKMV viruses, can inhibit the host RNA polymerase [98]. The examples discussed illustrate a broad conservation of SIE mechanisms across species.

Overall, numerous diverse mechanisms are utilized by phages that target different bacterial species to accomplish SIE. Many of these strategies are potentially translatable to *Pseudomonas* phages and provide reasonable insights to warrant future studies of SIE mechanisms.

**Table 1:** Summary table of all phages with known SIE mechanisms and their hosts that have been specifically discussed in this review. Also included is important information regarding the mechanisms they employ to accomplish superinfection exclusion, including the type of mechanism, known phage genes/proteins that are involved, and relevant citations.

Bacterial Host	Phage	SIE Mechanism(s)	Involved Phage Genes/Proteins	Reference
*Escherichia coli*	φ80	Receptor Inactivation	cor	[75]
*Escherichia coli*	N15	Receptor Inactivation	cor	[75]
*Escherichia coli*	T4	DNA Injection Inhibition	Imm, Sp	[78]
*Escherichia coli*	Lambda	Lytic Gene Transcription Inhibition	CI	[79,80,81,82,83,84,85,86,87,88,89]
		Cellular Energetics Modulation	RexA, RexB	
*Escherichia coli*	T7	Host RNA Polymerase Inhibition	Gp2, Gp0.7	[90,91,92,93,94,95]
*Pseudomonas aeruginosa*	Pf4	Receptor Inactivation	pVII, pVIII, pIII	[38,39,40,41,42,43,44,45]
*Pseudomonas aeruginosa*	D3112	Receptor Inactivation	Gp05	[13]
*Pseudomonas aeruginosa*	DMS3	Receptor Inactivation	Aqs1	[14]
*Pseudomonas aeruginosa*	vB_Pae_QDWS	Receptor Inactivation	Gp21	[15]
*Pseudomonas aeruginosa*	D3	Receptor Inactivation	Oac	[60,61]
*Pseudomonas aeruginosa*	φKMV	Host RNA Polymerase Inhibition	Rac	[96,97,98]
*Salmonella enterica*	P22	Receptor Inactivation	gtrA, gtrB, gtrC	[17,69,70,71]
		DNA Injection Inhibition	SieA	
*Shigella flexneri*	SF6	Receptor Inactivation	gtr	[72,73,74]
*Shigella flexneri*	SF101	Receptor Inactivation	oacB	[73,74]

## 6. Fitness Costs Associated with Prophage Induction and SIE Mechanisms

A growing body of evidence suggests that the presence of SIE mechanisms has a significant impact on the evolution and fitness of viral populations [99,100,101]. However, much less is known about how these mechanisms influence the viral host—a topic that is the primary focus of this review. We also suggest that the use of theoretical models to study viral populations in the aforementioned studies might be adapted to investigate the effects on host populations as well. It is possible that the activation of SIE mechanisms may have an unforeseen consequence for the bacterial host. As previously described, many mechanisms involve the modification of cell surface structures that serve important host functions. Even subtle modifications of these structures may result in significant fitness costs for the bacterial host [19].

The twitching motility of *P. aeruginosa* is an important contributor to virulence [102,103,104]. The loss of this motility, a direct consequence of T4P inactivation, is associated with reduced biofilm production, a limited ability to colonize tissues, and a compromised capacity for immune evasion. Notably, biofilm formation by *P. aeruginosa* plays a crucial role in antimicrobial resistance [22,105,106,107,108]. As such, a reduction in biofilm production leads to increased antimicrobial susceptibility. The diverse mechanisms through which *P. aeruginosa* prophages inactivate the T4P upon superinfection suggests the potential for these phages to incur fitness defects on their hosts.

The O-antigen serves as an essential component involved in bacterial immune evasion and complement resistance [109,110]. Consequently, any modification of the O-antigen by *Pseudomonas* phages may give rise to a variety of associated fitness costs for the host. Additionally, the O-antigen is linked to biofilm production and improves tissue adherence [111,112]. Any changes to the O-antigen structure could potentially affect the ability of *P. aeruginosa* to perform these functions, thereby influencing bacterial fitness, either improving or worsening it.

Lastly, the wide variety of mechanisms facilitating the prevention of viral DNA injection into the host may also impact bacterial fitness. The synthesis of proteins that localize to the cell membrane and prevent DNA injection requires essential resources, including amino acids, nucleotides, and replication machinery. Redirecting these essential bacterial resources to SIE mechanisms likely has a detrimental impact on bacterial growth and fitness. Additionally, the overall impact of these proteins on blocking DNA injection is unknown, and it is possible they might alter the bacterial membrane in certain locations, leading to unforeseen impacts on bacterial fitness.

Alternatively, certain aspects of the SIE mechanisms might improve bacterial fitness and contribute to a cooperative relationship between integrated prophages and their hosts [113]. The most obvious benefit SIE provides to a bacterial host is protection from lysis by other phages. Since SIE can prevent infection by similar, even purely lytic phages, the host can avoid death through the phage-mediated SIE mechanisms. Furthermore, SIE stabilizes prophages within the host, preventing prophage induction and excision. The protective prophage–host relationship provides an evolutionary advantage that supports such mutualistic benefits. Some of the benefits to the bacterial host facilitated by SIE could include increased biofilm production, resistance to antibiotics and environmental stressors, and enhanced virulence [114,115]. As discussed in other recent reviews, it is apparent that the fitness effects incurred by SIE are highly dependent on the specific mechanism of SIE and the environmental context that the host bacterium exists in [116].

## 7. Integrated Prophages as a Vector for the Dissemination of Virulence and Antimicrobial Resistance

While the process of SIE influences the fitness and behavior of a particular host or a single bacterial population, it is possible that it also plays a role in the spread of virulence and antimicrobial resistance across bacterial populations. Phages are excellent carriers of genetic material. They often serve as vehicles for the horizontal transmission of bacterial genes, including virulence and antimicrobial resistance genes [117]. Phages can facilitate genetic transfer via transduction, wherein an infecting phage packages bacterial DNA along with its own DNA and carries it on to its next host. Transduction leads to the efficient dissemination of these genes throughout bacterial populations and is an important driver of bacterial evolution. It has been shown that genetic material conferring resistance to important last-line antibiotics such as vancomycin and colistin have been isolated from phage fractions of wastewater [118]. Similar phage-mediated dissemination of antibiotic-resistance genes has been observed in poultry [119]. In addition to the accidental packaging of bacterial DNA, the induction of prophages has been demonstrated as a valid mechanism facilitating the inter- and intraspecies exchange and expansion of genetic material. As prophages are excised from their host and seek out new hosts, they can carry functional genes with them that confer to the new host upon integration [9,120]. This influence is evident by the high proportion of prophages containing virulence genes in important bacterial pathogens [117]. The complex interactions between SIE and the spread of virulence via phage vectors have not been extensively studied. Considering the importance of virulence dissemination for human health, it is reasonable to suggest that further studies should focus on the impact of these interconnected mechanisms.

The most characterized example of prophage-encoded virulence factors is found in the Shiga-toxin-producing phages of *E. coli*. These toxins are mainly produced upon induction of the prophage, leading to the lysis of the bacterium [121,122]. The mechanism facilitating Shiga toxin conversion is especially interesting because it provides a novel virulence strategy that drastically increases the severity of *E. coli* infections. For example, *E. coli* O157:H7 descended from an avirulent strain of *E. coli* and underwent lysogenic conversion, resulting in a highly virulent Shiga-toxin-producing strain [123,124]. Such a phenomenon of lysogenic conversion is significant for the maintenance of human health as it can lead to the rapid dissemination of virulence through a bacterial population [125,126,127]. This mechanism underscores the importance of screening potential phage therapy candidates for the presence of undesirable genes.

Surprisingly, phages rarely carry antimicrobial resistance (AMR) genes within their core genomes [128,129]. Instead, phages serve as vectors for horizontal gene transfer of bacterial-encoded AMR genes. Upon prophage induction, the phage genome often carries nearby bacterial genes with it as it exits the genome. Subsequently, during replication within the host and eventual lysis, the phage carries these nearby genes in a process called transduction as it seeks new hosts [130,131]. Such phage-mediated dissemination of genes provides an avenue for the rapid spread of AMR and virulence genes throughout bacterial populations. A relevant example of this is the transduction of resistance plasmids in methicillin-resistant *Staphylococcus aureus* (MRSA), where phages such as φ80α and φJB efficiently transfer tetracycline resistance and penicillinases [132]. This example demonstrates the clinical significance of phage transduction as a mechanism for the spread of virulence and AMR genes. Further complicating the issue, some phages are able to infect a more diverse range of hosts, and sometimes other species or even genera of bacteria [133,134]. Therefore, phage-mediated gene transfer significantly contributes to the emergence of new virulence, resistance mechanisms, and microbial evolution. However, there are notable research gaps regarding how specific SIE mechanisms might modulate these routes of undesirable gene transmission.

## 8. Discussion

### 8.1. Sublethal Concentrations of Antibiotics Facilitate Prophage Induction and SIE

Non-inhibitory concentrations of antibiotics can trigger integrated prophages to transition to their lytic state [135,136]. It is currently known that synthetic antimicrobials, such as quinolones and microbially-produced antibiotics such as mitomycin C, are potent inducers of prophages and do so via activation of the SOS response [137,138,139]. However, in addition to the SOS response, there are a variety of mechanisms by which environmentally produced antibiotics may mediate prophage induction, including the production of reactive oxygen species [140,141]. It is also known that many species of bacteria can produce antibiotics that instigate these mechanisms when they encounter other competing species [10]. Often, the concentrations that the antibiotic producers release are too low to directly kill bacteria, and the exact function of these molecules at sub-lethal concentrations is elusive [142,143]. However, at sub-effective concentrations, antibiotics may activate latent prophages in the competing bacterium. Such antibiotic-mediated activation may lead to bacterial killing through phage lysis or, as this review posits, impose fitness costs on the competing species, enabling the antibiotic producer to outcompete for resources.

### 8.2. Consequences of SIE: Host Fitness

**Figure 3:**
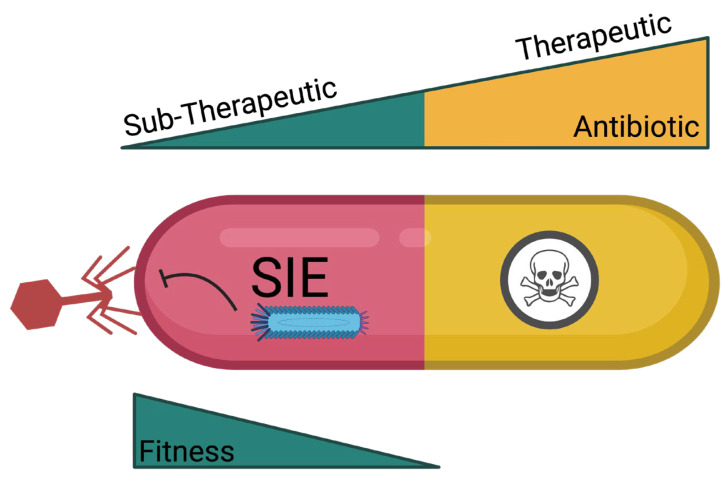
Sub-therapeutic concentrations of antibiotics can trigger prophage induction and activate superinfection exclusion, leading to compromised bacterial fitness.

Having specific integrated prophages in the genome is beneficial to the bacteria as prophages can trigger toxin production and increase virulence. However, under conditions of superinfection, SIE mechanisms could potentially alter the bacteria, resulting in reduced fitness. The effects on fitness may manifest as a reduced ability to colonize tissues, form biofilms, and maintain a normal growth rate. Therefore, it is possible that the forced induction of prophages using sub-inhibitory antibiotic concentrations may reduce the virulence and overall fitness of bacterial species that are difficult to kill through conventional methods (Figure 3). Such a phenomenon may not be limited to environmental bacteria but could also contribute to bacterial virulence in the presence of sub-inhibitory concentrations of antibiotics observed in humans and food animals undergoing antibiotic treatment or administration for growth promotion.

We hypothesize that the effects of prophage induction and the activation of specific SIE mechanisms on bacterial fitness might be underestimated due to the evident structural changes made in the bacterial surface components and the associated fitness costs linked to the inactivation of these structures. For example, numerous studies have demonstrated that sub-optimal growth conditions, such as high temperatures and extreme pH levels, lead to reduced biofilm production by *P. aeruginosa* [144,145]. However, these conditions are also known to induce prophages [6]. In the event of prophage induction, it is likely that their associated SIE mechanisms become active. A specific example that supports our hypothesis is the inactivation of the T4P phage receptor [12]. As the T4P is involved in twitching motility and therefore biofilm production, the possibility that the induction of prophages could impact biofilm reduction has not been fully considered. It is possible that a variety of studies showing the modulation of specific fitness and virulence phenomena have neglected to consider the impact of prophage induction and the consequent SIE.

### 8.3. Consequences of SIE: Spread of Virulence

As previously described, integrated prophages can serve as effective vectors for disseminating virulence genes within a population. As a component of the prophage lifestyle, SIE mechanisms are intertwined with the concept of virulence dissemination and provide an additional layer to the social interactions between these viruses and their hosts [113]. We hypothesize that SIE mechanisms play two contrasting roles in transferring virulence genes. The first possible role is the stabilization of virulence genes that are present within the prophage genome. SIE mechanisms protect integrated prophages from disruption by other competing phages in the environment. This stabilization allows the phage to remain integrated within the bacterial genome, facilitating the spread of virulence genes through vertical gene transfer or other modes of genetic exchange, such as conjugation. On the other hand, the second potential role of SIE in disseminating virulence genes within a bacterial population is warranted by stabilizing the integrated prophage regions and preventing prophage induction. Avoiding induction keeps the prophage from entering the lytic cycle, which would likely cause a massive release of phages potentially carrying virulence genes to rapidly spread across the entire population. Furthermore, prophages frequently carry with them virulence genes that are located near their integration sites, which might not be excised perfectly during prophage induction. If a given prophage is not induced, it cannot serve as a vector for the horizontal gene transfer of these adjacent genes to related bacteria.

### 8.4. Consequences of SIE: Clinical Utilization of Phages

SIE mechanisms not only prevent the infection by the same bacteriophage but also extend the protection against many similar phages, particularly those utilizing the same bacterial cell surface receptor. Such mechanisms could pose a potential challenge for phage therapy in strains that contain integrated prophages, as potent phages, like those used in phage therapy, might not infect bacteria because of the SIE mechanisms conferred by the integrated prophage. Contrary to this expectation, the use of phage therapy may still be beneficial in the treatment of these infections as the activation of SIE mechanisms can lead to the described fitness defects. Furthermore, it is possible that phage therapy would be more effective in combination with antibiotics when used to treat bacterial isolates containing prophages. While the phage–antibiotic synergy has been identified in numerous studies [146,147,148], the underlying mechanisms often rely on an evolutionary trade-off between mutual antibiotic and phage targets, such as efflux pumps [149], and implications of this synergism in prophage-containing isolates have yet to be explored. With the advancements in whole-genome sequencing technology, identifying prophage regions in bacterial genomes has become more accessible, even in clinical settings. Therefore, a crucial step prior to selecting phages for therapeutic use is to evaluate the presence of prophage regions that might influence the efficacy of lytic phages. SIE mechanisms pose an additional problem to acquired bacterial resistance. Similar to bacteria evolving to change their receptors and evade phage infection, many SIE mechanisms modify receptors to prevent infection. For example, utilizing phages that exclusively bind to the T4P in the treatment of *P. aeruginosa* PAO1 would be inefficient, given the role of the clearly described mechanism of SIE that inactivates that receptor. Instead, a more efficient therapeutic strategy would involve selecting a cocktail composed of a diverse set of phages capable of binding to different receptors, avoiding exclusion by these mechanisms.

## 9. Conclusions

In summary, the interplays among the integrated prophages, SIE mechanisms, and bacterial fitness underscore the intricate evolutionary relationship between bacteriophages and their bacterial hosts. The nuanced effects of SIE mechanisms on virulence dissemination and bacterial fitness open new avenues for research. Here, we have outlined the diversity of SIE mechanisms and their potential implications on single-host and population-wide dynamics. Our conclusions support that SIE mechanisms are not merely a competitive tool but a broader evolutionary strategy that shapes microbial ecology and has potential clinical applications. Understanding this dynamic relationship between phages and their host may lead to innovative therapeutic strategies where we could take advantage of SIE-mediated fitness defects and phage–antibiotic synergy. As bacterial resistance continues to challenge modern medicine, insights from these mechanisms might hold the key to developing more targeted and effective treatments in the future.
